# Factors affecting range of motion following two-stage revision arthroplasty for chronic periprosthetic knee infection

**DOI:** 10.1186/s43019-022-00162-2

**Published:** 2022-07-18

**Authors:** Doo-Yeol Kim, Young-Chae Seo, Chang-Wan Kim, Chang-Rack Lee, Soo-Hwan Jung

**Affiliations:** 1 Department of Orthopedic Surgery, Good Samsun Hospital, 356, Gaya-daero, Sasang-gu, Busan, 47392 Republic of Korea; 2 Department of Orthopedic Surgery, College of Medicine, Busan Paik Hospital–Inje University, 75, Bokji-ro, Busanjin-gu, Busan, 47392 Republic of Korea

**Keywords:** Knee, Arthroplasty, Periprosthetic joint infection, Treatment outcome, Range of motion

## Abstract

**Introduction:**

The purpose of this study was to evaluate factors that affect range of motion (ROM) following two-stage revision arthroplasty as a treatment for chronic periprosthetic knee infection.

**Materials and methods:**

A total of 98 patients diagnosed with chronic periprosthetic joint infection (PJI) following primary total knee arthroplasty between January 2009 and December 2019 and then underwent two-stage revision arthroplasty were reviewed retrospectively. Multiple regression analysis was performed to evaluate the factors that affect ROM after two-stage revision arthroplasty. ROM after two-stage revision arthroplasty was used as a dependent variable, while age at the time of surgery, ROM at PJI diagnosis, ROM after the first-stage surgery, the interval between the first-stage surgery and the second-stage surgery, whether a re-operation was performed before the second-stage surgery, culture results (culture negative or culture positive), and body mass index (BMI) were used as independent variables.

**Results:**

Multiple regression analysis (*R*^2^ = 0.843) revealed that among the independent variables, ROM (*β* = 0.604, *P* < 0.001) after the first-stage surgery, whether a re-operation was performed before the second-stage surgery (*β* =  − 8.847, *P* < 0.001), the interval between the first-stage surgery and the second-stage surgery (*β* = − 0.778, *P* = 0.003), and BMI (*β* =  − 0.698, *P* = 0.041) were associated with ROM after two-stage revision arthroplasty, the dependent variable.

**Conclusions:**

In two-stage revision arthroplasty for chronic periprosthetic knee infection, ROM after the first-stage surgery, whether a re-operation was performed before the second-stage surgery, the interval between the first-stage surgery and the second-stage surgery, and BMI were found to be factors that were associated with ROM after two-stage revision arthroplasty.

## Introduction

Periprosthetic joint infection (PJI) is one of the most serious complications that can occur after total knee arthroplasty (TKA). The incidence of PJI after primary TKA is reported to be between 1% and 3%, and PJI is one of the most significant factors causing early failure after TKA [1, 2]. Although the diagnosis of PJI is not easy, several groups have proposed criteria for this purpose, and these criteria are constantly being updated. PJI is largely divided into two categories, acute PJI and chronic PJI, depending on the time of onset after TKA and duration of symptoms [3–5]. Treatment of PJI requires surgical treatment along with the administration of appropriate antibiotics. There are a variety of surgical treatment options for PJI, including debridement, antibiotics, and implant retention (DAIR); one-stage revision arthroplasty; and two-stage revision arthroplasty. The gold standard for chronic PJI is considered to be two-stage revision arthroplasty, which is based on the concept of controlling infection and reimplanting the prosthesis through two surgeries [6–8]. The first-stage surgery includes removal of infected prosthesis, thorough debridement of infected soft tissue and bone, and insertion of antibiotic-impregnated cement spacers and/or beads, whereas the second-stage surgery includes removal of cement spacers and/or beads, debridement, and reimplantation with revision prosthesis [1].

A number of studies have reported relatively favorable outcomes with a re-infection rate of around 10–20% after two-stage revision arthroplasty [2, 9–13]. However, many earlier studies evaluated the outcomes of two-stage revision arthroplasty by focusing on the re-infection rate rather than on the functional outcomes. Functional outcomes, such as patient-reported outcomes and range of motion (ROM), are important factors in evaluating outcomes of TKA. In particular, post-operative ROM is considered to be an important factor that can affect the satisfaction rate after TKA [14–18]. It has been reported that various factors, such as age, pre-operative ROM, and body mass index (BMI), are factors that can affect ROM after primary TKA [19], and Ritter et al. [20] reported that these factors can also affect the postoperative ROM after revision TKA. However, Ritter et al. [20] did not mention the exact reason why the revision TKA was done and just how many surgeries was performed, which is a weakness of their study. To date, there appears to be a lack of research on factors affecting post-operative ROM after two-stage revision arthroplasty for PJI. Therefore, we focused our attention on this issue.

The purpose of this study was to evaluate the factors that affect two-stage revision arthroplasty ROM for chronic periprosthetic knee infection. We hypothesized that ROM after the first-stage surgery, the interval between the first-stage surgery and the second-stage surgery, the number of surgeries performed before the second-stage revision arthroplasty, and BMI would affect ROM after two-stage revision arthroplasty.

## Materials and methods

### Patients

This study was approved by the Institutional Review Board of our institution (IRB No, 2021-01-025). Patients who were diagnosed with chronic PJI following primary TKA between January 2009 and December 2019 and then underwent two-stage revision arthroplasty were reviewed retrospectively. The inclusion criteria of this study were: (1) patients who received primary TKA at our institution or other hospitals; (2) patients diagnosed with chronic PJI; and (3) patients who received two-stage revision arthroplasty using articulating-type antibiotic-impregnated cement spacers. The exclusion criteria were: acute post-operative or acute hematogenous PJI, or PJI after revision TKA, and those who underwent two-stage revision arthroplasty using static-type antibiotic-impregnated cement spacers. Given that the purpose of this study was to evaluate the factors affecting ROM after two-stage revision arthroplasty, patients who received a re-operation due to recurrence of deep infection or wound problem after two-stage surgery were also excluded.

**Fig. 1:**
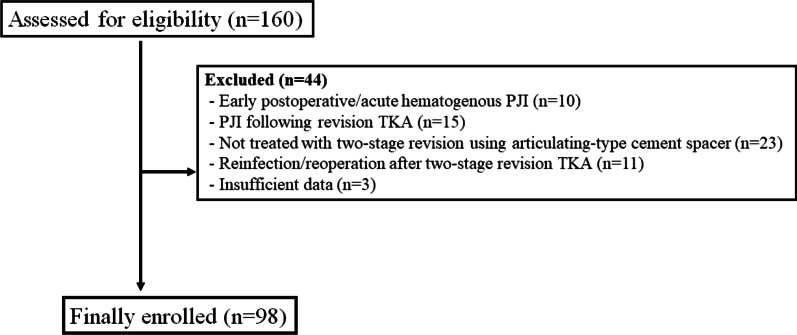
Patient eligibility flow chart.* PJI* Periprosthetic joint infection,* TKA* total knee arthroplasty

For the diagnosis of PJI, the criteria suggested by the Musculoskeletal Infection Society (MSIS) were used [21]. The major MSIS criteria are: (1) two positive periprosthetic cultures with phenotypically identical organisms; and (2) a sinus tract communicating with the joint. Minor MSIS criteria are: (1) elevated serum erythrocyte sedimentation rate (ESR) and C-reactive protein (CRP) (ESR > 30 mm/h and CRP > 10 mg/L); (2) elevated synovial fluid white blood cell (WBC) count (> 3000 cells/μL); (3) elevated synovial polymorphonuclear neutrophil (PMN) percentage (≥ 80%); (4) > 5 neutrophils per high-power field (HPF) in five HPFs (400×); and (5) a single positive culture. Patients who fell met at least one of the major criteria or three or more of the five minor criteria were diagnosed with PJI. Chronic PJI was defined as a case in which symptoms appeared > 4 weeks after primary TKA and which lasted > 3 weeks [4]. Two-stage revision arthroplasty was performed on all patients diagnosed with chronic PJI, with the exception of patients who did not consent to undergo surgical treatment or those who could not undergo surgery due to poor general health. The operation was performed by four experienced surgeons. Ninety-eight cases of chronic PJIs (98 patients) satisfying the inclusion and exclusion criteria were included in this study. The patient flow chart and the demographic data are summarized in Fig. 1 and Table 1, respectively.

**Table 1:** Patient demographic data

Patient demographic data	Values
Age (years)	68.9 ± 6.5
Sex, *n* (male/female)	21/77
BMI (kg/m^2^)	25.9 ± 2.8
Source of referral, internal/external, *n*	35/63
*Follow-up period (months)*	
After first-stage surgery	31.0 ± 8.0
After second-stage surgery	24.6 ± 7.9

Data in table are presented as the mean ± SD, unless indicated otherwise

*BMI* Body mass index,* SD* standard deviation

### Surgical technique and interim management

In the first-stage surgery, after the removal of the infected prosthesis and thorough debridement, articulating-type antibiotic-impregnated cement spacers and/or beads were inserted. These articulating-type cement spacers were made using a mold: 2 g of vancomycin was added and mixed per one bag (40 g) of polymethylmethacrylate cement powder. In some patients, 2 g of vancomycin and 1 vial of tobramycin (80 mg tobramycin) were mixed together. Tibial-side spacers were made to be thick enough to prevent hyperextension by evaluating the extension gap of the knee joint. Femoral and tibial side spacers were placed in the femur and tibia using 1 g of vancomycin with cement in the late doughy stage.

After the first-stage surgery, whether infection was controlled was evaluated using physical examination and laboratory tests, including ESR and CRP. However, no joint fluid analysis was performed routinely to determine whether infection was controlled. Parenteral antibiotics were administered for 4–8 weeks, and then parental antibiotics were changed to oral antibiotics or the administration of antibiotics was stopped, depending on the findings of the physical examination and the laboratory test results. The selection of antibiotics and duration of antibiotic treatment after surgery were based on the results of the culture test performed before or during surgery and the opinions of internal medicine or infection specialists. The second-stage surgery was considered when the laboratory test results remained normal for ≥ 2 weeks following the discontinuation of parenteral or oral antibiotics.

In the second-stage surgery, the cement spacers were removed and thorough debridement was performed using the same approach as used in the first-stage surgery. During surgery, five to six samples were collected to perform frozen biopsies, and the number of WBCs per HPF was counted by a pathologist. When > 10 WBCs per HPF were observed in > 2 samples, a new cement spacer was implanted. The constrained type of prosthesis was implanted when the infection was confirmed to be under control based on surgical findings and pathological examination. At the time of prosthesis implantation, 1 g of vancomycin was added and mixed per one bag of cement.

After the first-stage surgery, ROM exercise was allowed within the tolerable range, and partial weight bearing was allowed within the tolerable range while wearing a knee immobilizer. An ROM exercise using continuous passive motion (CPM) was started immediately after the second-stage surgery (remplantation), and weight bearing was also allowed.

### Evaluation

Information on age at the time of PJI diagnosis, sex, the amount of time that elapsed between TKA and the diagnosis of infection, culture results, ROM at PJI diagnosis, ROM after the first-stage surgery, ROM after the second-stage surgery, additional surgery done for superficial or deep infection, hematoma before the second-stage surgery, and re-infection were extracted the medical history and chart reviews. ROM was determined by a surgeon using a long-arm goniometer, measuring the passive ROM that the patient was able to move without pain. Flexion contracture and maximal flexion were measured, and ROM was defined as the difference between the maximal flexion and the flexion contracture [22]. Negative culture was defined as as the absence of a pathogen in the culture of the knee joint aspiration performed before surgery or in the joint fluid or tissue acquired during surgery.

### Statistical analysis

SPSS version 26.0 software (SPSS, IBM Corp., Armonk, NY, USA) was used for the statistical analysis. Statistical significance was set at *P* < 0.05. Multiple regression analysis was performed to evaluate the factors affecting ROM after two-stage revision arthroplasty. ROM after two-stage revision arthroplasty was used as a dependent variable, while age at the time of surgery, ROM at PJI diagnosis, ROM after the first-stage surgery, the interval between the first-stage surgery and the second-stage surgery, whether a re-operation was performed before the second-stage surgery, culture results (culture negative or culture positive), and BMI were used as independent variables.

## Results

The clinical outcomes of the patients are shown in Table 2. The mean ROM of patients at PJI diagnosis was 85.0° ± 32.6°; mean ROM after first-stage surgery was 75.1° ± 30.2°; and mean ROM after second-stage surgery was 105.9° ± 22.5°. Among the 98 patients included in this study, 50 patients were culture positive and 48 patients were culture negative. The mean length of the interval between first-stage surgery and second-stage surgery was 6.4 months (standard deviation: 3.8 months). he mean number of cases where additional surgery was performed before the second-stage surgery was 21 cases.

**Table 2:** Clinical outcomes

Clinical outcomes	Values
*Range of motion, °*	
At PJI diagnosis	85.0 ± 32.6
After first-stage surgery	75.1 ± 30.2
After second-stage surgery	105.9 ± 22.5
Positive/negative results, *n*	58/48
Interval (1st–2nd), months^a^	6.4 ± 3.8
No.(1st–2nd), *n*^b^	21

Data in table are presented as the mean ± SD, unless indicated otherwise

* PJI* Periprosthetic joint infection

^a^Interval between the first-stage surgery and the second-stage surgery

^b^Number of cases where additional surgery was performed before the second-stage surgery

The multiple regression analysis (*R*^2^ = 0.843; Table 3) revealed that ROM (*β* = 0.604, *P* < 0.001) after the first-stage surgery, whether a re-operation was performed before the second-stage surgery (*β* =  − 8.847, *P* < 0.001), the interval between the first-stage surgery and the second-stage surgery (*β* = − 0.778, *P* = 0.003), and BMI (*β* =  − 0.698, *P* = 0.041) were associated with ROM after two-stage revision arthroplasty. There was no variable with a variance inflation factor of ≥ 10, so it was considered that there was no multi-collinearity. The increase in ROM after the first-stage surgery was associated with an increase in ROM after two-stage revision arthroplasty; the history of re-operation before the second-stage surgery, an increase in the interval between the first-stage surgery and the second-stage surgery, and high BMI were correlated with a reduction in ROM after two-stage revision arthroplasty.

**Table 3:** Evaluation of factors the affect range of motion after two-stage revision arthroplasty (multiple regression analysis, stepwise method)

Dependent variable	Independent variables	Unstandardized coefficients		Standardized coefficients *β*	*P*
		*B*	SE (*B*)		
ROM after second-stage surgery (last FU ROM)^a^	ROM after first-stage surgery	0.604	0.033	0.811	< 0.001
	No. (1st–2nd),* n*^b^	− 8.847	2.306	− 0.162	< 0.001
	Interval (1st–2nd), weeks^c^	− 0.778	0.258	− 0.130	0.003
	Body mass index	− 0.698	0.337	− 0.085	0.041

*ROM* Range of motion,* SE* standard error

^a^Last FU ROM refers to follow-up range of motion (24.6 ± 7.9 months after second-stage surgery)

^b^Number of cases where additional surgery was performed before the second-stage surgery

^c^Interval between the first-stage surgery and the second-stage surgery

## Discussion

The main findings of this study were that ROM (*β* = 0.604, *P* < 0.001) after the first-stage surgery positively affected the ROM after reimplantation and that the interval between the first-stage surgery and the second-stage surgery (*β* =  − 8.847, *P* < 0.001), whether additional surgery was performed before the second-stage surgery (*β* = − 0.778, *P* = 0.003), and BMI (*β* =  − 0.698, *P* = 0.041) negatively affected ROM after reimplantation in patients who underwent two-stage revision arthroplasty as a treatment for chronic PJI following primary TKA.

The re-operation rate after primary TKA is reported to be about 5%, and various causes of TKA failure have been reported [23–25]. Several previous studies which analyzed the outcomes of aseptic or septic revision TKA reported significant improvements in function and pain after revision surgery. However, functional outcomes of aseptic revision TKA and septic revision TKA may be different. In revision surgery performed due to reasons other than infection, such as osteolysis and loosening, only one surgery is required, whereas two-stage revision arthroplasty performed on PJI patients requires at least two or more surgeries, and soft tissue problems also often accompany many of these cases. A review of several studies comparing the outcomes of aseptic and septic revision TKA revealed that, although controversial, septic revision TKA showed relatively poorer clinical outcomes, as shown in several published reports [26–30].

In two-stage revision arthroplasty for PJI, during the first stage surgery, articulating-type cement spacers are inserted rather than static-type cement spacers after the removal of the infected prosthesis, which is intended for greater recovery of ROM after the second-stage surgery. However, it is not easy for PJI patients to obtain sufficient ROM after two-stage revision arthroplasty, as least to the extent that there is no limitation in their daily activities. Therefore, we deemed it necessary to evaluate the factors affecting ROM after two-stage revision arthroplasty as a treatment for PJI. Previous studies reported that patient factors, such as causative diseases, degree of deformity, age, BMI, and pre-operative ROM, surgery-related factors, such as surgical approach and technique and component position and alignment, and other various factors, such as rehabilitation, may affect ROM after primary or revision TKA [19, 20, 31–33]. However, factors affecting ROM in two-stage revision arthroplasty for PJI may differ from those affecting ROM in conventional primary TKA.

ROM after the first-stage surgery, additional surgery before the second-stage surgery, and the interval between the first-stage surgery and the second-stage surgery are factors related only to two-stage revision arthroplasty which do not need to be considered in one-stage revision arthroplasty. In this study, the larger the ROM after the first-stage surgery was, the larger the ROM after two-stage revision arthroplasty. This relationship is thought to be similar to the results of several studies reporting that pre-operative ROM is an important factor affecting post-operative ROM after primary or revision TKA [20, 34–36]. Although articulating-type cement spacers were inserted to obtain a larger ROM, it is still not easy to achieve both knee joint stability and functional ROM simultaneously. If the condition of not getting enough ROM lasts for several months after the first-stage surgery due to various reasons, such as condition of the soft tissue and pain, there is a high possibility that sufficient ROM is not obtained even after the second-stage surgery due to stiffness of the knee joint. Therefore, we believe the spacers need to be made in appropriate thickness during the molding process of articulating-type cement spacers so that the flexion and extension gaps do not become so tight.

The results of the present study showed that in addition to ROM after the first-stage surgery, additional surgery before the second-stage surgery, the interval between the first-stage surgery and the second-stage surgery, and BMI also negatively affected ROM after two-stage revision arthroplasty. Additional surgery before the second-stage surgery is believed to be related to the limited ROM after the second-stage surgery, given that it may also be related to soft tissue problems or long-term immobilization. The interval between the first-stage surgery and the second-stage surgery—that is, the timing of the second-stage surgery—is determined by various conditions, such as the patient's general health condition, soft tissue condition, laboratory test results (including ESR, CRP, etc.), and the infection control status [1]. The delay in the timing of the second-stage surgery may indicate poor patient health, poor soft tissue conditions, or poor infection control in the patient. Similar to the history of additional surgery before the second-stage surgery, these conditions may lead to reduced ROM after the second-stage surgery. Specifically, a prolonged interval between the first-stage surgery and the second-stage surgery without obtaining functional ROM after the first-stage surgery is believed to have an increased negative effect on ROM after the second-stage surgery.

BMI is not a factor that a surgeon can control to get a larger post-operative ROM. Various results have been reported on the effect of BMI on ROM after TKA. There are several published reports of a high BMI negatively affectig ROM after primary TKA [37, 38].

Given that two or more surgeries are generally performed for the treatment of chronic PJI, we hypothesized that ROM after the first-stage surgery, additional surgery before the second-stage surgery, and the interval between the first-stage surgery and the second-stage surgery would be factors that could affect ROM after two-stage revision arthroplasty. Our results support this hypothesis. Taking these findings into account, with the aim to increase the possibility of obtaining functional ROM after the second-stage surgery, we recommend that surgeons determine the appropriate thickness of cement spacers during the first-stage surgery to obtain sufficient ROM, thereby reducing the need for additional surgery as much as possible through thorough debridement and the use of appropriate antibiotics, and not to delay the timing of the second-stage surgery.

There are several limitations to this study. First, this study was a retrospective study targeting a small number of cases. Second, there might be slight differences in surgical procedure and post-operative assessment because surgery was performed by four different surgeons. Third, this study was a short-term follow-up study and included patients who were followed up for > 1 year after the second-stage reimplantation. Although some patients who had not been followed up for > 2 years after the second-stage surgery were included in this study, the purpose of this study was to evaluate factors affecting ROM, not patient-reported outcomes or radiologic complications after surgery. Therefore, it is believed that there was enough time to recover ROM at the time point when 1 year has elapsed after the second-stage surgery and that there would be no significant difference in the ROMs after 1 year and after 2 years following the operation. Fourth, in many of the cases included in this study, primary TKA was performed at other hospitals. Therefore, it was not possible to evaluate the ROM before the onset of PJI. Fifth, implants from different manufacturers were used for two-stage revision arthroplasty for the patients included in this study. Sixth, the degree of maximal knee flexion and the knee flexion contracture were not analyzed separately in this study. If such an analysis could be done for each patients, we may have obtained additional meaningful results. Finally, clinical scores, which are another meaningful indicator for the functional outcome, were not assessed in this study because the records were missing for some patients.

## Conclusions

In two-stage revision arthroplasty for chronic periprosthetic knee infection, ROM after the first-stage surgery, the interval between the first-stage surgery and the second-stage surgery, whether a re-operation was performed before the second-stage surgery, and BMI were found to be factors that were associated with ROM after two-stage revision arthroplasty.

## Data Availability

Not applicable.
